# Factors Predicting Spontaneous Closure of Patent Ductus Arteriosus in Very Low Birth Weight Neonates

**DOI:** 10.7759/cureus.68241

**Published:** 2024-08-30

**Authors:** Kiran S Depala, Shaaista Budhani, Miheret Yitayew, Janardhan Mydam

**Affiliations:** 1 School of Medicine, Vanderbilt University, Nashville, USA; 2 Neonatology, John H. Stroger, Jr. Hospital of Cook County, Chicago, USA; 3 Neonatology, Children’s Hospital of Richmond at Virginia Commonwealth University, Richmond, USA

**Keywords:** very low birth weight infant, urine output, hemodynamically significant pda, neonates, patent ductus arteriosus

## Abstract

Background

Patent ductus arteriosus (PDA) is a relatively rare condition among neonates in the United States, estimated at approximately 1 in every 2,000 births. Managing hemodynamically significant patent ductus arteriosus (hsPDA) in very low birth weight (VLBW) neonates remains a challenge. This study aims to explore and report on the spontaneous closure of hsPDA in VLBW infants in a clinical setting.

Methods

We conducted a retrospective review of VLBW infants born from 2006 to 2014 at our institution. Infants included in the study were diagnosed with hsPDA via echocardiogram in the first week of life. We divided our population into two study groups: 1) those with PDA closure before discharge without medical/surgical treatment (spontaneous closure) and 2) those with closure by medical/surgical treatment. Relevant baseline data and PDA-related parameters were extracted from the medical records.

Results

A total of 108 infants were included in the study; 52 experienced spontaneous PDA closure, and 56 required treatment. Of the baseline characteristics, gestational age and mode of delivery differed significantly between the two groups. Within the adjusted model, cesarean section (CS) (OR: 0.18; 95% CI: 0.06-0.55), average pre-diagnosis partial pressure of carbon dioxide (PCO2) (OR: 0.92; 95% CI: 0.86-0.98), and pre-diagnosis daily fluid intake (OR: 0.96; 95% CI: 0.94-0.99) were associated with increased odds of spontaneous closure.

Conclusion

In our study of VLBW infants, several variables were associated with spontaneous hsPDA closure. Studies with larger sample sizes are much needed and have the potential to clinically impact the outcomes of neonates living with this relatively rare condition.

## Introduction

The appropriate management of patent ductus arteriosus (PDA) in very low birth weight (VLBW) infants is a moving target. After decades of an aggressive approach, conservative strategies are now trending [1-4]. The incidence of PDA in infants <28 weeks of gestation ranges from 34 to 70%, and the rate of spontaneous closure of PDA ranges between 40-67% [2]. Recent evidence suggests that early closure of a PDA may not yield long-term benefits or improve mortality rates [5,6], and the ductus arteriosus closes spontaneously in a substantial number of VLBW infants before discharge [7,8]. However, a persistent PDA with a sizeable left to right shunt can create pulmonary hypertension with increased pulmonary vascular resistance (PVR) and the phenomenon of diastolic steal, increasing risks of bronchopulmonary dysplasia (BPD), necrotizing enterocolitis (NEC), renal dysfunction, and intraventricular hemorrhage (IVH) [9]. Declines in neurological outcomes and increased mortality have also been reported in the literature.

Persistent PDA closure can be achieved with cyclooxygenase (COX) inhibitors like indomethacin and ibuprofen, acetaminophen, or interventions such as surgical ligation or percutaneous closure; however, all these solutions could be hazardous for VLBW infants. Randomized control trials of pharmacological and surgical treatments to close HSPDA in preterm infants have not shown long-term benefits. COX inhibitors increase the risk of NEC, reduce renal blood flow, and decrease cerebral blood flow [10-12], while ligation has both short-term and long-term adverse effects [11,13]. A tool to predict which VLBW infants are more likely to experience spontaneous PDA closure would spare some infants from the risks of these treatments. Similarly, the ability to predict which infants will develop clinically significant PDA allows for timely treatment, sparing these infants from the complications of PDA. The success of pharmacologic closure may also be improved with an earlier age at first treatment [14-16].

Several variables have been identified as independent risk factors for persistent ductal patency. Older gestational age is possibly the most consistently documented factor independently associated with spontaneous closure [7,8,17,18]. Other potentially predictive variables include B-type Natriuretic Peptide (BNP) levels [19], larger duct size (measured by echocardiogram) [17,20], birth weight [20,21], use of steroids [18], factors related to platelet function [22,23], and respiratory distress syndrome [18,24].

The need for new clinical or laboratory markers to predict the course of a PDA in order to limit intervention to a select group of infants is well-recognized [25]. Therefore, we designed this retrospective study to look for associations between several demographic and clinical variables and PDA closure in VLBW infants. Our goal is to identify and quantify potential predictors of spontaneous closure in this population.

This article was previously posted to the preprint service on Research Square on August 1, 2023.

## Materials and methods

Data and study population

We conducted a retrospective case-control study of 108 VLBW (<1500g) infants born from January 2006 to December 2014 at John H. Stroger Jr. Hospital of Cook County, Chicago, IL, United States. Inclusion criteria included VLBW infants with echocardiographically diagnosed moderate to large PDA. Specifically, an infant was identified to have a hemodynamically significant PDA based on a ductus arteriosus diameter exceeding 1.5 mm, a left atrium (LA)/Aorta ratio of ≥1.4, and a requirement for ≥40% oxygen due to PDA (corroborated by radiologic indications of increased lung blood flow and not arising from other respiratory issues such as respiratory distress syndrome or sepsis) [26].

Inclusion criteria

**Figure 1:**
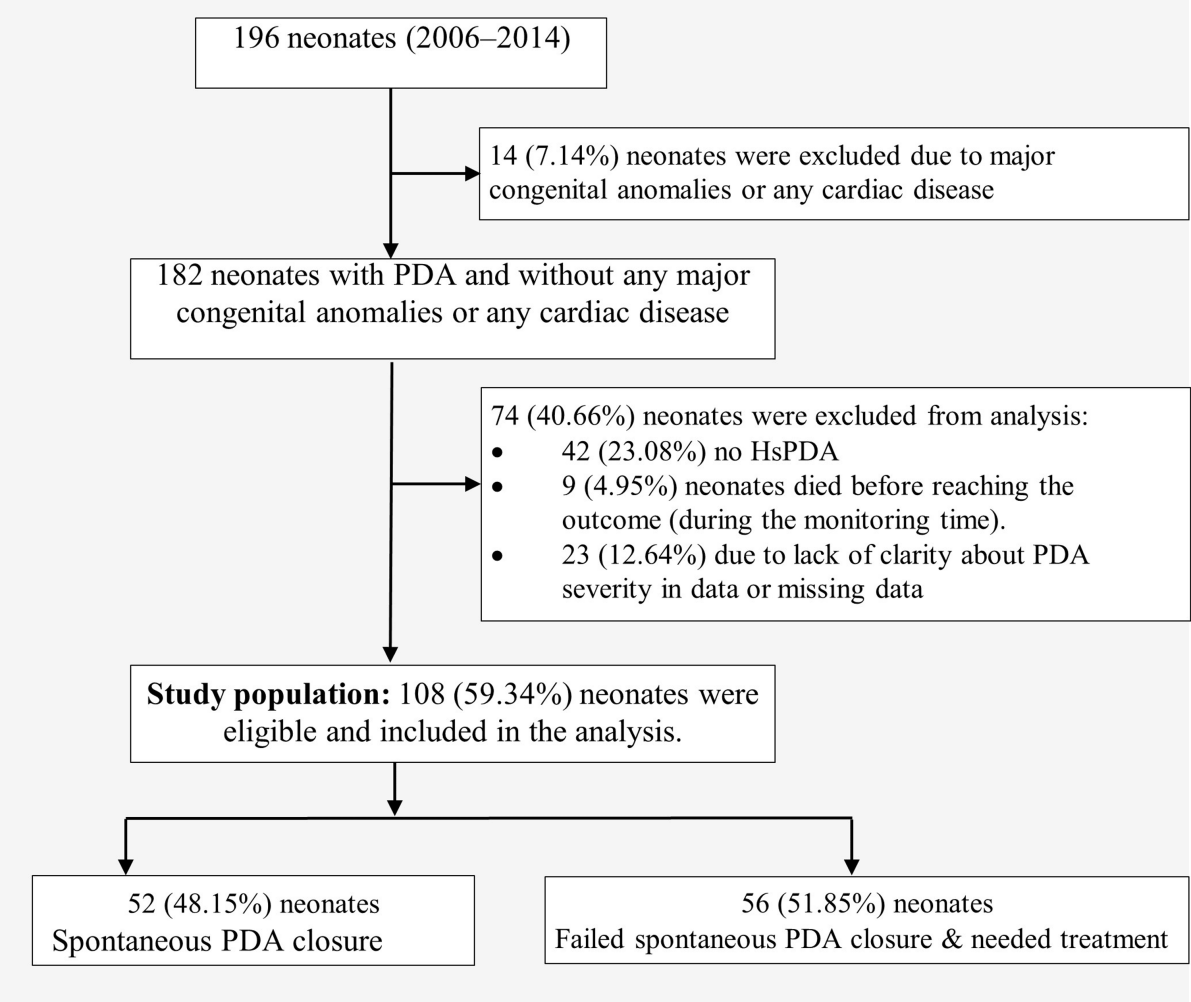
Exclusion criteria and study population breakdown. Selection and classification flowchart for VLBW infants in retrospective study. HsPDA: Hemodynamically significant patent ductus arteriosus; VLBW: Very low birth weight.

We included only VLBW infants with echo-diagnosed moderate to large PDA. A total of 108 infants (52 with spontaneous PDA closure without treatment and 56 who needed pharmacological or surgical treatment) were included in our study (Figure 1). The decision to treat the PDA was at the discretion of the attending clinician, often influenced by factors such as gestational age and infant weight.

Exclusion criteria

Before analysis began, we gathered data from 196 neonates. Exclusions were neonates with major congenital anomalies, PDAs accompanied by other cardiac conditions, early fatalities before PDA outcome determination, and cases with unclear or missing PDA severity data. Of these infants, 182 met the inclusion criteria; 14 infants were excluded due to major congenital anomalies or PDA with any other cardiac anomalies/diseases. Of the 182 infants, 74 were further excluded due to 9 deaths, 42 instances of non-hemodynamically significant patent ductus arteriosus (HsPDA), and 23 cases with unclear PDA severity data or missing data.

Demographic and clinical variables

Our variables included demographic, baseline, and clinical characteristics of our patient population, as well as PDA-related clinical parameters. We extracted the following baseline data from the medical records: maternal age, race/ethnicity, sex of the infant, gestational age, duration of rupture of membranes (ROM), use of antenatal steroids, presence of chorioamnionitis, use of vasopressors, multiple gestation, birth weight, 1-minute, 5-minute, and 10-minute Apgar scores, surfactant treatment, admission temperature, magnesium use, mode of delivery, endotracheal tube (ETT) use, and resuscitation medications. The clinical variables included in the analysis were sodium (Na), hematocrit, hemoglobin, potential of hydrogen (pH), carbon dioxide (CO2), serum bicarbonate (HCO3), base excess, fluid goal, and urine output. In our study, "fluid goal" refers to the volume of fluid an infant receives in milliliters per kilogram per day. Infants were divided into two groups according to 1-minute Apgar (≤5 and >5), 5-minute Apgar (≤5 and >5), 10-minute Apgar (≤5 and >5), and ROM duration (≤18 h and >18 h).

Statistical analysis

We divided our study population into two groups for statistical analysis: 1) those with spontaneous PDA closure before discharge without pharmacological or surgical intervention, and 2) those requiring such interventions due to critical clinical conditions such as worsening ventilatory settings, frequent desaturations, increased FiO2 requirements, and signs of organ dysfunction. Our primary outcome variable was spontaneous closure of PDA as determined by echocardiogram during the study period. To explore potential associations between our variables and the outcome of interest, we performed chi-square tests for categorical variables, independent sample t-tests for normally distributed continuous variables, and Wilcoxon-Mann-Whitney U tests for non-normally distributed continuous variables. Additionally, we performed time trend analysis from day one to the diagnosis day of statistically significant repeated measures.

We then performed multivariable logistic regression analysis to identify variables significantly associated with spontaneous PDA closure. A p-value of <0.05 was considered statistically significant within our analysis. We also performed sensitivity and specificity analysis for the significant predictors from the regression analysis. The optimal cut-off point for a clinical parameter was determined by identifying the value that yielded the minimum difference between sensitivity and specificity. All statistical analyses were conducted using SAS 9.4 software (SAS Institute, Inc., Cary, NC, USA). In the multivariable analysis, cut-off points for sensitivity and specificity of significant variables were determined and benchmarks identified using SAS 9.4 software; both the highest sensitivity cut point and an optimal (balanced) cut point that maximizes the combined sensitivity and specificity values are also reported.

## Results

A total of 108 infants were analyzed in the study; 52 (48.1%) experienced spontaneous PDA closure and 56 (51.9%) required treatment. Demographic and baseline characteristics of the study population are described in Table 1. Gestational age (GA), admission temperature, and mode of delivery significantly differed between the two groups. GA was significantly higher in infants with spontaneous PDA closure compared with those who required treatment (26 weeks vs 25 weeks; P=0.017), while admission temperature was significantly lower among the infants with spontaneous PDA closure (36.70°C vs 36.80°C; P=0.02). Although the difference in temperatures across both groups shows minimal variability, the consistency of this variability between the two groups and the significance of the p-value are notable. We also found the mode of delivery to be associated with spontaneous PDA (P-value = 0.003); the rate of spontaneous PDA closure was significantly higher for infants born by cesarean section (CS) compared with those born by vaginal delivery (VD) (61.82% versus 32.69%, P=0.003).

**Table 1:** Demographic characteristics of the study population (n = 108). *Values are rounded to the nearest integer. ROM: Rupture of membranes; VD: Vaginal delivery; CS: Cesarean section; ETT: Endotracheal tubes. Categorical variables: Data are displayed by count (n) and percentage (%). Chi-square tests were conducted to compare percentage distributions between groups with spontaneous PDA closure and those needing treatment due to failed spontaneous PDA closure. Continuous variables (normally distributed): Data are presented by mean and standard deviation. Independent samples t-tests were conducted to compare the mean scores between spontaneous PDA closure and failed spontaneous PDA closure needing treatment. Continuous variables (not normally distributed): Data are displayed by median and range (min-max). Wilcoxon Mann Whitney U tests were performed to compare the median values between spontaneous PDA closure and failed spontaneous PDA closure needing treatment. The distribution of continuous variables was compared using Wilcoxon Mann Whitney U tests.

Demographic characteristics	Spontaneous PDA closure N = 52 (48.15%)	Failed Spontaneous PDA closure and needed treatment N = 56 (51.85%)	P-value
Maternal Age (Years) (median (range))	25.50 (15.00-45.00)	24.00 (13.00-41.00)	0.12
Race/Ethnicity (n (%))			
White	3 (5.77)	3 (5.36)	0.49
Black	37 (71.15)	38 (67.86)	
Hispanic	10 (19.23)	15 (26.79)	
Female infants (n (%))	26 (50.98)	22 (39.29)	0.22
Gestational age (weeks) (median (range))	26.00 (23.00-34.00)	25.00 (21.00-32.00)	0.02
ROM duration (Hrs) (median (range))	0.00 (0.00-672.00)	0.00 (0.00-360.00)	0.39
ROM duration (Hrs) (median (range))			0.42
<= 18 hours	28 (70.00)	38 (77.55)	
> 18 hours	12 (30.00)	11 (22.45)	
Antenatal Steroids (n (%))	44 (84.62)	45 (80.36)	0.56
Chorioamnionitis (n (%))	5 (9.62)	9 (16.36)	0.3
Vasopressors use (n (%))	22 (44.00)	23 (41.07)	0.76
Multiple Gestations (n (%))	8 (15.38)	6 (10.71)	0.47
Birth Weight (gm) (mean (SD))	875.04 (290.88)	806.70 (274.95)	0.21
APGAR score at 1st min (n (%))			0.18
0-5	32 (64.00)	41 (75.93)	
>5	18 (36.00)	13 (24.07)	
APGAR score at 5th min (n (%))			0.44
0-5	9 (18.00)	13 (24.07)	
>5	41 (82.00)	41 (75.93)	
APGAR score at 10th min (n (%))			0.08
0-5	5 (35.71)	2 (9.09)	
>5	9 (64.29)	20 (90.91)	
Surfactant use (n (%))	46 (88.46)	54 (96.43)	0.18
Admission Temp (°C) * (median (range))	36.70 (32-38)	36.80 (33-38)	0.02
Magnesium (n (%))	31 (60.78)	31 (57.41)	0.73
Mode of delivery (n (%))			< 0.01
VD	17 (33.33)	35 (62.50)	
CS	34 (66.67)	21 (37.50)	
ETT use (n (%))	39 (75.00)	46 (82.14)	0.37
Resuscitation medications use (n (%))	8 (15.38)	3 (5.36)	0.09

Statistically significant differences in key clinical characteristics between our two groups are shown in Table 2, which displays the Day 1 clinical characteristics of the study population classified by outcome. Hematocrit (HCT) (44.6±8.7 vs 39.4±8.7; P=0.003), hemoglobin (15.3±3.1 vs 13.4±2.9; P=0.002), and fluid goal (90.0 vs 100.0; P=0.033) differed significantly between the two outcome groups.

**Table 2:** Clinical characteristics of the study population. For normally distributed variable(s), 'mean (SD)' is presented along with the p-value from the t-test. For non-normally distributed variable(s), 'median (range)' is presented along with the p-value from the Wilcoxon–Mann–Whitney U test.

Variable	Spontaneous PDA closure N = 52 (48.15%)	Failed Spontaneous PDA closure and needed treatment. N = 56 (51.85%)	P-value
Sodium (mean (SD))	136.27 (4.51)	137.41 (3.34)	0.14
Hematocrit (mean (SD))	44.56 (8.70)	39.37 (8.65)	0.003
Hemoglobin (mean (SD))	15.27 (3.13)	13.38 (2.94)	0.002
pH (median (range))	7.32 (7.00-7.44)	7.31 (6.99 - 7.49)	0.97
Carbon dioxide (median (range))	40.48 (23.17-64.00)	39.77 (25.4-86.30)	0.4
Serum bicarbonate (median (range))	21.20 (10.60-27.20)	20.80 (12.70-26.20)	0.82
Base excess (median(range))	-4.00 (-21.00-1.00)	-5.00 (-15.00-0.00)	0.48
Weight (median (range))	790.00 (345.00-1430.00)	717.50 (415.00-1420.00)	0.28
Fluid goal/received (median (range))	90.00 (70.00-136.00)	100.00 (70.00-120.00)	0.033
Urine output (median (range))	4.20 (0.70-10.50)	3.53 (0.96-9.90)	0.35

Within Table 3, we report the pre-diagnosis measurements for select clinical parameters averaged over a two to three-day period prior to PDA diagnosis. There were statistically significant differences between the following clinical parameters between the two outcome groups: pH (7.32 vs. 7.29, P=0.03), carbon dioxide (CO2) (45.2±10.1 vs. 53.5±8.1; P=0.004), bicarbonate (HCO3) (22.34±4.53 vs. 24.81±5.06; P=0.009), fluid goal (118.1±21.6 vs. 133.3±13.7; P= <0.001), and urine output (3.16±0.93 vs. 3.55±0.87; P=0.03). A steep increase in CO2, fluid goal, and HCO3 were associated with failed spontaneous closure, while a steep decrease in urine output was associated with spontaneous closure.

**Table 3:** Pre-diagnostic hematological and biochemical characteristics of the study population. For normally distributed variable(s), 'mean (SD)' is presented along with the p-value from the t-test. For non-normally distributed variable(s), 'median (range)' is presented along with the p-value from the Wilcoxon–Mann–Whitney U test.

Variable (average of 2-3 days prior-diagnosis measurements)	Spontaneous PDA closure N = 52 (48.15%)	Failed Spontaneous PDA closure and needed treatment. N = 56 (51.85%)	P-value
Sodium (mean (SD))	137.9 (4.92)	138.0 (4.55)	0.93
Hematocrit (median (range))	40.81 (29.17-71.00)	40.40 (28.00-56.67)	0.46
Hemoglobin (median (range))	13.94 (9.92-24.10)	13.50 (9.50-19.27)	0.42
pH (median (range))	7.32 (7.06-7.43)	7.29 (7.20-7.38)	0.03
PaCO2mean (SD))	45.22 (10.05)	53.51 (8.00)	<0.0001
PAO2 (median (range))	52.00 (33.00-144.00)	40.88 (26.75-70.00)	<0.0001
Serum bicarbonate (mean (SD))	22.34 (4.53)	24.81 (5.06)	0.009
Base excess (median (range))	-2.47 (-18.00-8.50)	-0.14 (-10.00-10.83)	0.12
Weight ( median (range))	833.58 (342.50-1751.67)	753.33 (425.00-1403.33)	0.15
Fluid goal (mean (SD))	118.1 (21.62)	133.3 (13.70)	<0.0001
Urine output (mean (SD))	3.16 (0.93)	3.55 (0.87)	0.03
Blood urea nitrogen (median (range))	25.00 (15.00-70.00)	34.50 (10.00-120.00)	0.3
Serum creatinine (median (range))	0.90 (0.40-1.40)	0.90 (0.50-1.60)	0.85

The results of the logistic regression analysis are presented in Table 4. Within the adjusted model, mode of delivery, average CO2, average partial pressure of oxygen (PO2), and average fluid goal were found to be associated with spontaneous PDA closure. Infants delivered vaginally were 82% less likely to experience spontaneous PDA closure (OR=0.180; 95% CI: 0.059-0.553) compared with those delivered by CS. For every single-unit increase in pre-diagnosis average CO2, the odds of spontaneous PDA closure decreased by 8% (OR=0.919; 95% CI: 0.862-0.978), while for every single-unit increase in pre-diagnosis average PO2, the odds of spontaneous PDA closure increased by approximately 5% (OR=1.047; 95% CI: 1.004-1.093). Additionally, for every single unit increase in pre-diagnosis average fluid goal, the odds of spontaneous PDA closure decreased by 3% (OR=0.97; 95% CI: 0.94-0.99).

**Table 4:** Multivariable logistic regression analysis to predict the likelihood of spontaneous PDA closure. In this analysis, only those predictor variables which are significant in simple logistic regression and do not have a multicollinearity problem have been considered.

Variable	OR (95% CI)	P-value
Hematocrit (HCT) on day 1	1.057 (0.992-1.126)	0.0854
Mode of delivery (Ref=Vaginal delivery )	0.180 (0.059-0.553	0.0028
2-3 days pre-diagnosis average Carbon dioxide	0.919 (0.862-0.978)	0.0081
2-3 days pre-diagnosis average PO2	1.047 (1.004-1.093)	0.0322
2-3 days pre-diagnosis average fluid goal	0.967 (0.939-0.996)	0.0257

Further results are detailed in Table 5, which illustrates the calculated sensitivity, specificity, positive predictive value (PPV), negative predictive value (NPV), positive likelihood ratio (LR+), and negative likelihood ratio (LR−) for each cut-off value of several clinical parameters. For day one hematocrit (HCT), the optimal cut-off value was 43, yielding a sensitivity of 63.5%, specificity of 67.3%, PPV of 64.7%, and NPV of 66.1%, with an LR+ of 1.94 and an LR− of 0.54. For average bicarbonate (HCO3), the optimal cut-off was 22, with sensitivity, specificity, PPV, NPV, LR+, and LR− of 50.0%, 37.5%, 42.6%, 44.7%, 0.80, and 1.33, respectively. The optimal cut-offs for average CO2, average PO2, average fluid goal, and average urine output were 48, 46, 129 ml/kg/day, and 3.3 ml/kg/hr, respectively. Their associated diagnostic accuracy metrics are listed in Table 5.

**Table 5:** Diagnostic accuracy of predictive biomarkers for spontaneous PDA closure. Sensitivity, specificity, Positive Predictive Value and Negative Predictive Value are presented in percentage (%) along with their 95% CI. HCT: Hematocrit; HCO3: Bicarbonate; CO2: Carbon dioxide; PO2: Partial pressure of oxygen; PDA: Patent ductus arteriosus. *High sensitivity cut point #Optimal/ideal cutoff point: This value is considered the optimal/ideal cutoff point for which the difference (sensitivity – specificity) is minimum. $High specificity cut point

Risk factor	Cutoff point	Sensitivity (95% CI)	Specificity (95% CI)	Positive Predictive Value (95% CI)	Negative Predictive Value (95% CI)	Positive Likelihood Ratio (95% CI)	Negative Likelihood Ratio (95% CI)
Mode of delivery		66.67 (53.73-79.60)	0.6250 (49.82-75.18)	61.82 (48.98-74.66)	67.31 (54.56-80.06)	1.78 (1.08-2.48)	0.5333 (0.30-0.77)
Average CO2	40*	71.15 (58.84,83.47)	3.57 (0.00,8.43)	40.66 (30.57,50.75)	11.76 (0.00,27.08)	0.74 (0.62,0.89)	8.08 (1.94,33.63)
	48#	32.69 (19.94,45.44)	26.79 (15.19,38.38)	29.31 (17.60,41.02)	30.00 (17.30,42.70)	0.45 (0.29,0.68)	2.51 (1.57,4.03)
	64$	5.77 (0.00,12.11)	91.07 (83.60,98.54)	37.50 (3.95,71.05)	51.00 (41.20,60.80)	0.65 (0.16,2.57)	1.04 (0.93,1.15)
Average PaO2	34*	96.15 (90.93,100)	16.07 (6.45,25.69)	51.55 (41.60,61.49)	81.82 (59.03,100)	1.15 (1.01,1.30)	0.24 (0.05,1.06)
	46#	69.23 (56.69,81.78)	67.86 (55.63,80.09)	66.67 (54.09,79.24)	70.37 (58.19,82.55)	2.15 (1.41,3.28)	0.45 (0.29,0.71)
	71$	15.38 (5.58,25.19)	98.21 (94.75,100)	88.89 (68.36,100)	55.56 (45.77,65.34)	8.62 (1.12,66.55)	0.86 (0.76,0.97)
Average Fluid Goal	104*	71.15 (58.84,83.47)	2.00 (0.00,5.88)	43.02 (32.56,53.49)	6.25 (0.00,18.11)	0.73 (0.61,0.87)	14.42 (1.98,105.17)
	129#	34.62 (21.68,47.55)	30.00 (17.30,42.70)	33.96 (21.21,46.71)	30.61 (17.71,43.52)	0.50 (0.33,0.75)	2.18 (1.37,3.48)
	158$	1.92 (0.00,05.66)	96.00 (90.57,100)	33.33 (0.00,86.68)	48.48 (38.64,58.33)	0.48 (0.05,5.14)	1.02 (0.95,1.09)

**Figure 2:**
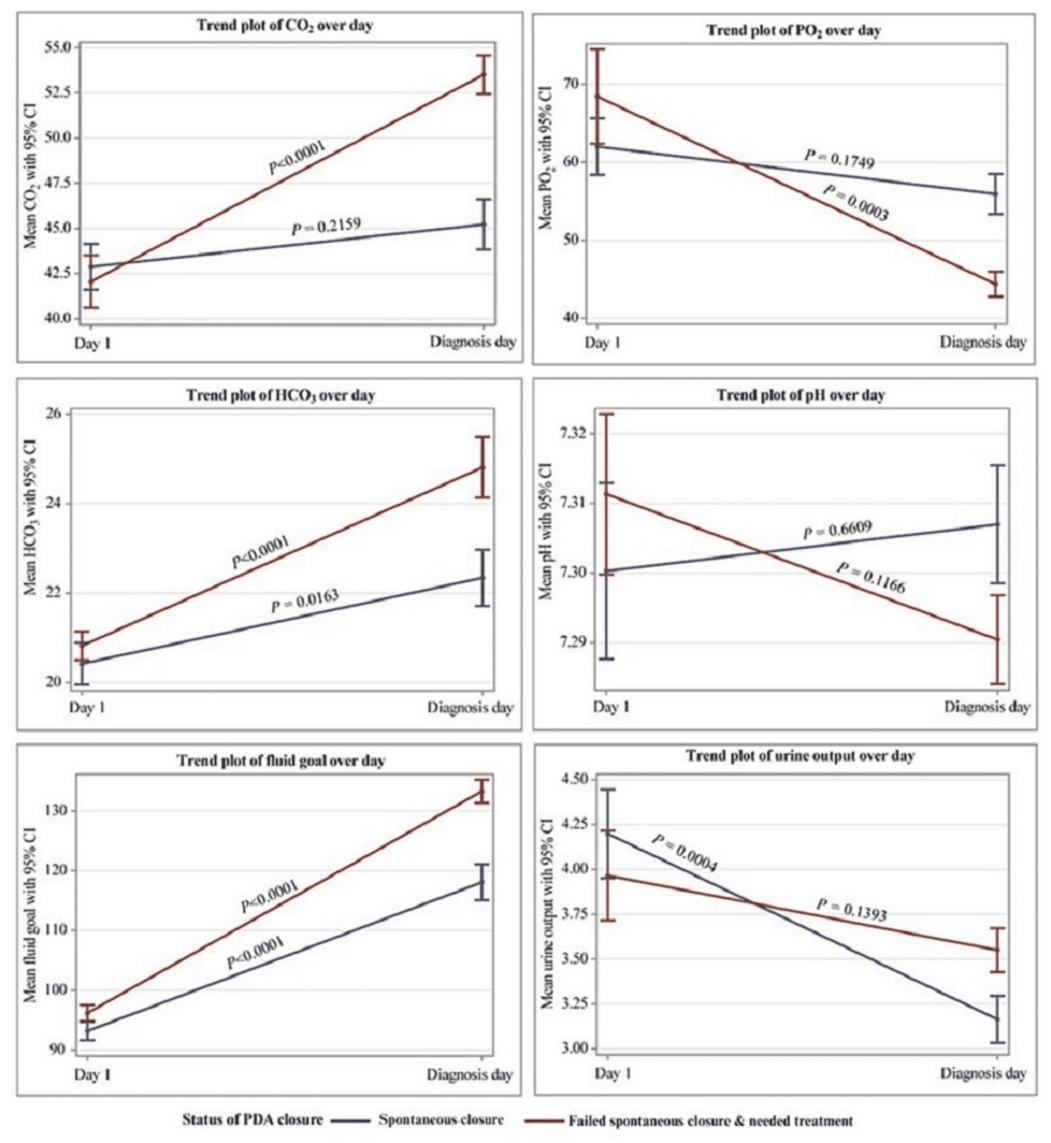
Trend plots of CO2, PO2, HCO3, pH, fluid goal, and urine output on day 1 compared to 2-3 days before diagnosis.

Time trend analysis, as shown in Figure 2, revealed significant differences in selected clinical parameters during the days leading up to PDA diagnosis. In both outcome groups, CO2 increased from day one to diagnosis day; however, CO2 increased more rapidly in the group that failed spontaneous PDA closure and required treatment. Notably, CO2 in the failed-closure group was lower than in the spontaneous closure group on day one but higher on the day of diagnosis. Average fluid goal and bicarbonate increased and average urine output decreased from day one to diagnosis day in both outcome groups.

**Figure 3:**
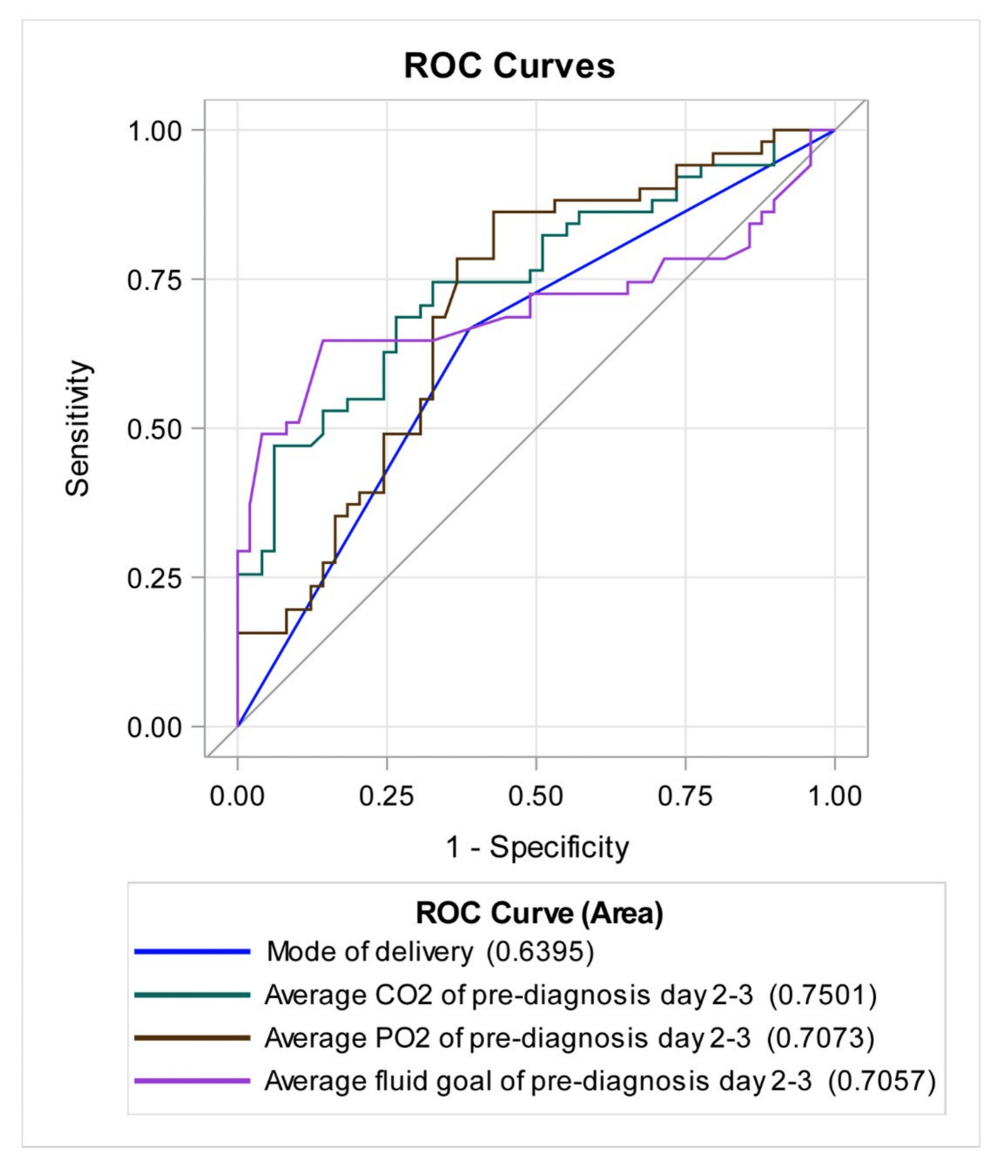
Receiver operating characteristic (ROC) curve analysis for mode of delivery, day 1 hematocrit, average CO2 from pre-diagnosis days 2-3, average PO2 from pre-diagnosis days 2-3, and average fluid goal from pre-diagnosis days 2-3.

We calculated the ROC curve to evaluate the strength of the model, with results shown in Figure 3. Figure 3 displays the ROC curves of the significant variables from the multivariable logistic regression prediction model. The average PCO2 has the highest area under the curve (75%), followed by average PO2 and average fluid goal, both approximately 70%.

## Discussion

In this retrospective study, we analyzed data from 108 VLBW infants diagnosed with moderate-to-large PDA in the first week of life. Within our adjusted logistic regression model, we found that cesarean delivery, lower average pre-diagnosis partial pressure of carbon dioxide (PCO2), higher average PO2, and lower pre-diagnosis daily fluid goal were associated with spontaneous closure.

The spontaneous closure rate in our population was 48.1%, which is considerably lower than several studies reporting spontaneous closure rates ranging from 71% to 85% [4,8,21]. However, unlike our study, which included only infants with moderate-to-large and HsPDAs, previous studies included infants regardless of initial PDA size. This gap in closure rates aligns with findings by Thankavel PP et al. [17], who demonstrated that ductus arteriosus diameter on the third day of life was a significant predictor of spontaneous closure in VLBW infants. Other studies have also shown that this diameter is inversely associated with spontaneous closure [26,27]. Nearly half of our population with confirmed moderate-to-large ductal openings did close spontaneously, underscoring the importance of selective treatment to avoid unnecessary treatment risks.

Many earlier studies have observed associations between gestational age and spontaneous closure in VLBW infants, the younger the gestational age, the less likely the PDA will close on its own [7,8,17,18,21]. In our study, the median gestational age of infants with spontaneous PDA closure was greater than that of infants who failed to close, at a statistically significant level, complementing the existing body of evidence. Our finding that infants delivered vaginally were less likely to close spontaneously is consistent with Ngo S et al., who found that infants delivered vaginally were more likely to be diagnosed with PDA than those delivered by CS [28]. To our knowledge, no study has reported a significant difference in admission temperature between spontaneous closures and closures requiring intervention, although the temperature difference was statistically significant (36.7°C vs 36.8°C; P<0.02). Further research is necessary to fully understand its clinical significance.

In our study, there was a significant difference in several clinical variables between the two groups under investigation. Initial univariate analysis revealed that infants who closed spontaneously had higher pH, higher PO2, lower PCO2, lower HCO3, lower fluid goal, and lower urine output (each averaged over the 2-3 days prior to PDA diagnosis), and higher hemoglobin and hematocrit (HCT) on the first day of life (Tables 2 and 3). In a 2014 study, Chen YY et al. also found an association between hemoglobin and PDA, concluding that lower hemoglobin levels after birth increase the risk of PDA in VLBW infants [24]. They postulated that since high oxygen tension is important for ductal constriction [29], a higher concentration of hemoglobin may influence closure by increasing oxygen levels [24]. Finally, in our study, an acidic environment (lower pH) was found to be associated with failure of spontaneous closure, similar to findings from Steiner M et al., who reported that lower pH values in the first 48 hours of life were associated with the need for ligation [30]. A further study by Kalis NN et al. found that higher HCO3 levels were associated with indomethacin resistance [31].

Our finding of an association between a lower fluid goal and spontaneous closure is consistent with the results of the study by Bell EF et al., who, upon reviewing several randomized clinical trials, found that fluid restriction reduces the risk of PDA in preterm infants [32]. Kahvecioglu D et al. also reported that infants with a lower median fluid intake were more likely to close spontaneously [23], while Stephens BE et al. found high fluid intake on days two and three of life to be associated with PDA independent of other factors [33]. In a paper addressing the complexities of PDA management, Benitz WE notes that although fluid received is associated with ductal closure, the optimal fluid goal for VLBW infants with PDA has not been determined [5]. Our study supports this result; however, the lack of an evidence base demonstrates a clear need for further research into this potentially clinically significant area.

Noori S et al. found that the incidence of HsPDA is higher with lower O2 saturation limits [34], while Inomata K et al. determined that a lower oxygen saturation target range in the first 72 hours of life was a risk factor for PDA closure failure [35]. This is consistent with our observation that infants with higher PO2 were more likely to close spontaneously; arterial PO2 and O2 saturation are directly proportional or positively associated. Similarly, Steiner M et al. found that infants with PDA who ultimately required ligation had significantly lower oxygen saturation in the first five days of life [30]. Dani C et al. reported associations between the highest FiO2 and PDA closure in both their 2008 and 2013 studies [7,36]. Our finding of an association between high PO2 and ductal closure, combined with our finding of an association between hemoglobin and closure, supports the assertion by Chen YY et al. that hemoglobin influences closure by its capacity to deliver oxygen [24].

Our study sought to explore the associations between key variables and spontaneous PDA in a neonate sample. Despite the above key results, we also observed some limitations. Our study period was limited to infants born between 2006 and 2014 due to data constraints from our institution's PDA registry. Despite this, the incidence of PDA remains constant, and its management in VLBW infants continues to be a topic of debate. The ability to predict spontaneous closure in PDA in VLBW infants has also remained elusive and requires clarity. Because of this, we believe the application of our results in current clinical practice remains relevant and timely. Our population size was limited and from a single center, which limits generalizability. The demographic and socioeconomic characteristics of the patient population and the subjects analyzed at our safety net hospital may not be representative of the United States at large. Additionally, we did not measure platelets or use platelet counts in our analyses. The broad GA range of our included infants is another limitation, as it covers weeks that might not be typically linked with HsPDA. Furthermore, while certain variables showed associations with spontaneous ductus arteriosus closure, their individual clinical utility is questionable due to lower sensitivity and specificity. However, we postulate that when these variables are amalgamated with other established parameters, they might offer more comprehensive and clinically relevant insights. Overall, we believe that our study offers insights into some of the factors associated with spontaneous closure, but that further research is needed to fully understand how best to clinically predict and treat the condition.

## Conclusions

A variable that could reliably predict the natural course of a PDA is considered the 'holy grail' of neonatology, and the quest is ongoing. We found that CD, lower average pre-diagnosis PCO2, higher average PO2, and lower pre-diagnosis daily fluid goal are associated with spontaneous closure. While many of our results are supported by previous research, future investigations will need to utilize larger sample sizes if we aim to use study results as predictors of spontaneous closure and/or to inform guidelines for the treatment of this relatively rare condition.
